# Health related quality of life measured by SF-36: a population-based study in Shanghai, China

**DOI:** 10.1186/1471-2458-8-292

**Published:** 2008-08-19

**Authors:** Rui Wang, Cheng Wu, Yanfang Zhao, Xiaoyan Yan, Xiuqiang Ma, Meijing Wu, Wenbin Liu, Zheng Gu, June Zhao, Jia He

**Affiliations:** 1 Department of Health Statistics, Second Military Medical University, Shanghai, PR China; 2 R&D Medical affairs, AstraZeneca Pharmaceutical Co., Ltd, Shanghai, PR China

## Abstract

**Background:**

Health related quality of life (HRQL) is a research topic that has attracted increasing interests around the world over the past two decades. The 36-item Short Form (SF-36) is a commonly used instrument for measuring HRQL. However, the information on Chinese adults' quality of life is limited. This paper reports on the feasibility of using the Mandarin version of SF-36 to evaluate HRQL in the population of Shanghai, China.

**Methods:**

A total of 1034 subjects were randomly sampled using a stratified multiple-stage sampling method in Shanghai. Demographic information was collected, and SF-36 was used to measure HRQL.

**Results:**

Internal reliability coefficients were greater than 0.7 in six of the eight SF-36 dimensions, except social function and mental health. Intraclass correlation coefficients ranged from 0.689 to 0.972. Split-half reliability coefficients were higher than 0.9 in five SF-36 dimensions. Validity was assessed by factor analysis and correlation analysis. Our results were basically in accordance with the theoretical construction of SF-36. The average scores of most SF-36 dimensions were higher than 80. The primary influencing risk factors of HRQL included chronic diseases, age, frequency of activities, and geographical region, which were identified using multivariate stepwise regression.

**Conclusion:**

Overall, HRQL in the population of Shanghai is quite good. The Mandarin version of SF-36 is a valid and reliable tool for assessing HRQL.

## Background

Conception of health has been changed with the development of medicine and medical sciences since 1970's. Health is defined as a dynamic state of human wellbeing characterized by a physical, mental, and social potential which satisfies the demands of a life corresponding to age, culture, and personal responsibility, and not merely the absence of disease or infirmity. Health related quality of life (HRQL) is an individual's satisfaction or happiness with the dimensions of life insofar as they affect or are affected by "health" as defined above. HRQL has been introduced to assess people's health status. To date, a number of questionnaires have been developed to evaluate HRQL, and the 36-item Short Form Health Survey (SF-36) is the most commonly used one.

SF-36 was developed from the Medical Outcomes Study or RAND Health Insurance Experiment [1]. It is a short-form derived from a larger 149-item instrument and is more precise than its predecessor, SF-20 [2]. SF-36 has been proven useful in monitoring population health, estimating the burdens of different diseases, monitoring outcome in clinical practice, and evaluating medical treatment effects. It has been translated into many languages with its content examined cross cultures [3-6]. In mainland China, the Mandarin SF-36 has been used in some surveys to assess the quality of life of the population with special chronic diseases [7,8]. However, the surveys on general populations were conducted only in Sichuan and Hangzhou [9,10].

In this study, we aimed at (1) testing the reliability and validity of the Mandarin version of SF-36; (2) assessing health related quality of life in the population of Shanghai, China; and (3) evaluating risk factors that may significantly influence HRQL. The study was approved by the Second Military Medical University Ethics Committee.

## Methods

### Sample

Shanghai is the biggest city in eastern China. It consists of 18 districts and 1 county that are geographically divided into 3 strata as urban, suburban, and rural regions. Using stratified multiple-stage sampling method, 4 residential areas in the urban region, 2 villages in the rural region, and 3 residential areas in the suburban region were selected following the sequence of district-block-residential area. A total of 1200 subjects older than 18 years of age were randomly sampled from those areas and 1034 subjects actually answered the questionnaires. The sample had 362 respondents from the Huangpu district, 336 from the Pudong district and 336 from the Songjiang district. The overall response rate was 86.17%. In order to analyze the reliability of the results, 10% of the total number of the respondents (i.e., 120 respondents) were randomly selected to take a retest by filling in the questionnaires again 2–7 days after the baseline test. At last 113 subjects took the retest. All respondents signed a written informed consent before participation.

### Questionnaire

The questionnaire included general information and Mandarin version of SF-36 [See additional file 1: Questionnaire-bilingual]. General information was collected on age, sex, resident region, nationality, marital status, educational level, current job, family monthly income, height, weight, tobacco use, alcohol use, and frequency of activities. Body Mass Index (BMI) was calculated from height and weight. Since the current WHO BMI criterion is suitable for Caucasians rather than Asians, additional BMI categories for Asian populations are recommended by WHO [11]. Many Asian countries have also developed their own criterions, such as Japan [12]. We used the Chinese BMI criterion as follows: underweight was defined as BMI lower than 18.5 kg/m^2^, healthy weight as BMI from 18.5 to 23.9 kg/m^2^, overweight as BMI from 24 to 27.9 kg/m^2^, and obesity as BMI of 28 or more kg/m^2^[13]. In addition, respondents were asked whether they had been diagnosed by physicians with the following chronic conditions: hypertension, ischemic heart disease, cerebrovascular disorder, diabetes, chronic obstructive pulmonary disease (COPD), asthma, renal disorder, liver disorder, rheumatoid arthritis, osteoarthritis, anxiety, and depression, and at which age the disease had been first diagnosed. The use of medicine at the time of the interview was also recorded.

The Mandarin version of SF-36 was translated from the IQOLA SF-36 Standard UK Version 1.0 by the experts of Zhejiang University, China. Its reliability and validity have been tested in the survey of Hangzhou, the capital of Zhejiang Province, southeast of Mainland China [10]. It was a brief self-administered questionnaire that generated assessment scores across eight dimensions of health: physical function (PF), role limitations due to physical problems (RP), bodily pain (BP), general health (GH), vitality (VT), social function (SF), role limitations due to emotional problems (RE), mental health (MH), and one single item dimension on health transition. The SF-36 dimensions can also be divided into two categories: Physical Component Summary (PCS) and Mental Component Summary (MCS), which represent the physical functioning and wellbeing, and emotional wellbeing, respectively.

### Field work

The survey was conducted from November 2005 to January 2006, using a self-finished interview method. Respondents filled in the questionnaires by themselves in their household or in local resident committees. The interviewers were social workers on the site who provided explanation without inducement on any unclear questions. Ten percent (10%) of the respondents had a repeatable accuracy check by filling in the questionnaires by themselves again a week later. The interviewers who interviewed the same respondent in the second time were different from the initial ones for the purpose of quality control. The performance of the interviewers was oversaw and coordinated by supervisors who examined questionnaires for any errors and ensured the quality of the survey. A valid questionnaire was the one that had been audited and signed by a supervisor. Both supervisors and interviewers were trained by the experts from Changhai Hospital and the epidemiologists from Second Military Medical University (SMMU).

### Data Management

All valid questionnaires were doubly input into the database by two independent professional data processors in the Department of Health Statistics of SMMU using software EpiData 3.1. Both manual checking and computer checking were conducted to find discrepancies.

In the Pudong District, a total of 112 respondents' questionnaires were withdrawn from the statistical analysis due to one facilitator's failure to adhere to the study protocol. Three questionnaires from the Huangpu District were excluded because more than 80% items were missing. Therefore, after the data checking and validation, 919 effective questionnaires were used for data analyses in this study. Of the 113 respondents who agreed to be re-interviewed, 14 questionnaires were rejected because they were not completed in line with the study protocol, resulting in 99 questionnaires for the retest analysis.

The missing values in the SF-36 dimensions were imputed as follows: if 50% or more items in one dimension were completed, the mean value of the completed items was used to impute the missing values. If more than 50% of the items were missing, the dimension score was excluded from the statistical analysis. In our survey, the item response rates were actually quite high. The average item response rate of the general information was 98.81%; the average response rate of the 36 items in SF-36 was 99.67%, ranging from 98.80% to 99.89%.

### Statistical analysis

The items and dimensions in SF-36 were constructed using the Likert method of summated ratings. The raw score of each of the eight SF-36 dimensions was derived by summing the item scores, and converted to a value for the dimension from 0 (worst possible health state measured by the questionnaire) to 100 (best possible health state). The raw score was then re-calculated across the dimension as follows:

$$\text{Transformed scale}=\left[\frac{\text{Actual raw score }-\text{lowest possible raw score }}{\text{Possible raw score range }}\right]\times 100$$

The PCS and MCS scores were calculated using the standard scoring algorithms [14-17].

The SF-36 questionnaire was evaluated by reliability and validity. Split-half reliability was computed by correlating the scores of the odd half with those of the even half in each dimension of SF-36. Test-retest reliability was assessed by the differences between test and retest scores using a paired-sample t test. It was further assessed by intraclass correlation coefficient (ICC). A questionnaire with ICC value larger than 0.7 was usually considered satisfactory [18]. Internal consistency of the SF-36 items was assessed by Cronbach's α coefficient. A Cronbach's α value of 0.7 or higher was generally considered to be sufficient to demonstrate internal consistency [18]. Construction validity was assessed by correlation analysis and factor analysis using principal component analysis and quartimax rotation. Factor loadings larger than 0.50 within a particular dimension were considered to support its factor construction. The cumulative variance proportion was used to indicate the contributions of the factors [19].

Statistical Analysis System (SAS) 9.1.3 and SPSS 10.0 were used for analyzing the survey data. Student t test, analysis of variance, and multivariate stepwise regression were applied to investigate the impact of various risk factors on quality of life.

## Results

### Sample characteristics

A total of 919 subjects were utilized in the statistical analyses, including 509 female and 410 male. The age of all subjects ranged from 18 to 77 years with a mean age of 47 ± 13 years.

### Reliability

#### 1. Split-half Reliability Analysis

Five of the eight SF-36 dimensions (i.e., PF, RP, BP, VT, and RE) had the split-half reliability coefficient higher than 0.9, while the other three dimensions (i.e., GH, SF and MH) had the coefficient lower than 0.7. The lowest split-half reliability coefficient (0.368) was observed for the SF dimension (Table 1).

**Table 1:** Reliability and correlation of the SF-36 dimensions.

		**Reliability**				**Correlation**			
			
**Dimension**	**Item amount**	**Split-half reliability**	**Test-retest difference mean**	**ICC**	**Cronbach's α**	**Correlations between dimensions and items inside**	**Correlations between dimensions and items outside**	**PCS**	**MCS**
PF	10	0.909	0.153†	0.964	0.862	0.257–0.922	0.164–0.564	0.756	0.340
RP	4	0.974	-1.020†	0.735	0.950	0.869–0.905	0.108–0.655	0.634	0.425
BP	2	0.904	-1.224†	0.817	0.863	0.837–0.976	0.063–0.363	0.656	0.177
GH	5	0.593	0.010†	0.952	0.818	0.716–0.785	0.123–0.624	0.756	0.517
VT	4	0.927	-0.867*	0.972	0.785	0.644–0.868	0.155–0.624	0.533	0.690
SF	2	0.368	-0.225†	0.689	0.308	0.662–0.687	0.067–0.322	0.326	0.508
RE	3	0.967	0.000†	0.898	0.951	0.877–0.952	0.080–0.614	0.351	0.599
MH	5	0.647	-0.204†	0.817	0.691	0.596–0.801	0.095–0.579	0.266	0.877

* P-value = 0.034, † *P-value *> 0.05

#### 2. Test-Retest Reliability Analysis

The absolute mean differences between the test and retest scores ranged from 0.000 to 1.224. The paired-sample t test indicated that the difference between the test and retest scores was not statistically significant for seven of the eight dimensions, except the VT dimension (*p-value *< 0.05). The one-week ICC ranged from 0.689 (the SF dimension) to 0.972 (the VT dimension) for the eight SF-36 dimensions (Table 1).

#### 3. Cronbach's α Analysis

The internal reliability of SF-36 was measured by Cronbach's α coefficient, which ranged from 0.308 (the SF dimension) to 0.951 (the RE dimension) for the eight SF-36 dimensions (Table 1).

### Validity

#### 1. Factor Analysis

The results of the factor analysis were described in details in the previous study [20], and are briefly summarized here. Eight factors plus health transition item were created with a cumulative variance proportion of 71.25%. The RE and BP dimensions were perfectly in accordance with the theoretical construction of SF-36. Other items were basically correlated with the factors as expected.

#### 2. Correlation Analysis

Spearman correlation analysis showed that the correlations between the dimensions and items inside were higher than those between the dimensions and items outside. It was evident that the PF, RP, BP, and GH dimensions were correlated with PCS, while the VT, SF, RE, and MH dimensions were correlated with MCS. Among the eight SF-36 dimensions, PF was the best measure of physical health and MH was the best measure of mental health. In contrast, MH and BP were the poorest measures of the physical and mental components, respectively (Table 1).

### HRQL

Table 2 showed the normative values of the SF-36 dimension scores by age and sex groups. The quality of life was reduced with increasing age. Female had lower scores than male in almost all subgroups, but in some subgroups female did report a better mental health. The SF-36 dimension scores were compared among different Chinese populations (Table 3): (1) every dimension score of the Shanghai population was higher than those of both Hangzhou population and American Chinese [10,21]; (2) the Shanghai population had higher scores than those of the Sichuan population in the RP, BP, SF, RE, and MH dimensions, but were similar to the Sichuan population in the PF, GH, and VT dimensions [9]; and (3) the SF-36 dimension scores of Hong Kong, Taiwan, American, and Canadian were lower than those of the Shanghai population in six of the eight dimensions, except GH and PF [22-25].

**Table 2:** Normative values of the SF-36 dimension scores by age/sex in the Shanghai population [Mean (SD)]

**Age**	**Sex**	**PF**	**RP**	**BP**	**GH**	**VT**	**SF**	**RE**	**MH**	**PCS**	**MCS**
18–29	Female	96.76(9.69)	95.96(19.13)	96.71(12.30)	81.83(16.70)	83.75(14.87)	94.85(13.18)	97.06(17.02)	88.76(9.85)	55.50(5.61)	57.02(5.02)
	Male	97.32(7.84)	98.91(6.69)	98.23(6.74)	84.04(15.76)	84.06(14.88)	95.83(10.43)	98.07(12.63)	88.06(11.09)	56.42(2.83)	56.87(4.49)
30–39	Female	96.15(6.78)	96.54(16.46)	94.45(14.13)	72.00(18.13)	75.92(13.26)	93.95(11.99)	96.92(17.40)	85.05(12.48)	54.53(3.76)	55.13(5.07)
	Male	96.85(7.77)	97.83(14.74)	99.09(4.46)	77.78(16.02)	76.74(16.47)	95.92(9.15)	100.00(0.00)	84.17(14.99)	55.82(3.14)	55.36(5.44)
40–49	Female	89.77(14.64)	93.98(21.31)	92.98(16.14)	69.36(16.67)	68.09(18.60)	95.49(9.28)	95.99(19.18)	78.19(16.61)	53.10(5.71)	53.36(6.44)
	Male	90.48(18.24)	92.72(24.66)	96.13(11.39)	68.15(21.35)	70.29(19.03)	93.15(15.47)	94.55(21.84)	78.44(13.81)	53.41(6.49)	53.05(6.07)
50–59	Female	87.62(13.35)	91.44(26.23)	94.56(13.21)	64.79(18.49)	68.61(18.16)	94.09(11.68)	93.12(24.41)	80.89(14.50)	51.91(5.54)	53.88(6.67)
	Male	88.44(15.48)	93.20(23.84)	94.90(14.32)	64.87(17.59)	69.20(18.03)	94.60(12.53)	94.13(22.82)	79.71(16.23)	52.41(5.48)	53.67(6.66)
60-	Female	79.14(18.20)	90.96(27.49)	90.00(17.24)	58.18(19.41)	65.60(19.42)	92.77(12.37)	91.97(25.28)	80.58(15.05)	49.00(6.67)	54.22(6.76)
	Male	83.18(13.63)	92.80(23.93)	91.58(17.04)	61.76(17.46)	68.94(16.77)	92.99(12.23)	95.96(17.06)	81.88(14.43)	50.19(5.80)	54.97(6.22)

Total	Female	89.05(14.56)	93.16(23.53)	93.75(14.67)	67.89(19.21)	70.97(18.38)	94.26(11.58)	94.55(21.83)	81.85(14.64)	52.51(5.92)	54.41(6.33)
	Male	90.55(15.01)	94.50(21.30)	95.70(12.49)	69.88(19.66)	72.78(18.23)	94.33(12.66)	95.85(18.84)	81.64(14.75)	53.36(5.62)	54.45(6.11)

**Table 3:** The SF-36 dimension scores of different Chinese populations [Mean (SD)]

Dimension	Shanghai		Hangzhou^10 ^	Sichuan^9 ^	Hong Kong^22 ^	Taiwan^23 ^	American Chinese^21 ^	American^24 ^	Canadian^25 ^
	(N = 919)		(N = 1688)	(N = 2249)	(N = 2410)	(N = 1191)	(N = 156)	(N = 2474)	(N = 9423)
								
	Mean(SD)	95%CI							
PF	89.7(14.8)	88.8, 90.7	82.2(19.8)	90.6(15.4)	91.8(12.9)	92.6(11.5)	79.4(23.4)	84.2(23.3)	85.8(20.0)
RP	93.8(22.6)	92.3, 95.2	81.2(33.6)	79.5(34.7)	82.4(31.0)	83.6(28.9)	67.5(37.3)	81.0(34.0)	82.1(33.2)
BP	94.6(13.8)	93.7, 95.5	81.5(20.5)	85.6(18.4)	84.0(21.9)	82.4(16.8)	62.3(21.9)	75.2(23.7)	75.6(23.0)
GH	68.8(19.4)	67.5, 70.0	56.7(20.2)	69.6(21.3)	56.0(20.2)	67.5(18.2)	58.8(22.7)	72.0(20.3)	77.0(17.7)
VT	71.8(18.3)	70.6, 73.0	52.0(20.9)	70.3(17.1)	60.3(18.7)	65.3(15.2)	59.0(20.3)	60.9(21.0)	65.8(18.0)
SF	94.3(12.1)	93.5, 95.1	83.0(17.8)	86.9(17.3)	91.2(16.5)	79.4(16.0)	75.1(22.7)	83.3(22.7)	86.2(19.8)
RE	95.1(20.6)	93.8, 96.5	84.4(32.4)	76.5(38.5)	71.7(38.4)	71.3(37.0)	61.2(43.7)	81.3(33.0)	84.0(31.7)
MH	81.8(14.7)	80.8, 82.7	59.7(22.7)	72.7(16.8)	72.8(16.6)	68.4(14.7)	63.9(20.4)	74.7(18.0)	77.5(15.3)

### Risk factors

Analysis of variance was first used to select the risk factors of HRQL. Region, gender, current job, age, current marital status, highest level of education, total income of family per month, frequency of activities, BMI, and chronic diseases were found influencing at least one dimension of SF-36. Multivariate stepwise regression was then applied using the SF-36 dimensions as the dependent variables and the risk factors mentioned above as the independent variables. The statistical significance level was set at 0.15 for both inclusion and exclusion of the independent variables in the stepwise process. The results indicated that the risk factors were different among the eight SF-36 dimensions (Table 4). Chronic diseases were evidently the most common risk factor reducing the scores of all SF-36 dimensions (*p-value *< 0.05). Its influence was relatively strong because its standardized regression coefficient was the largest one (in absolute value) for most of the SF-36 dimensions. Increasing age reduced quality of life in the PF, BP, GH, and VT dimensions, while manual work led to worse scores in the GH, VT and SF dimensions (*p-value *< 0.05). In contrast, high family income led to a good quality of life in the RE and MH dimensions. Similarly, frequent activities increased the quality of life in the PF, GH, VT, and MH dimensions (*p-value *< 0.05). The quality of life was improved with increasing education level in the RP dimension (*p-value *< 0.05). The unmarried had better quality of life in the VT and MH dimension (*p-value *< 0.05), but its standardized regression coefficients were quite low. The impact of gender and BMI was not statistically significant (*p-value *> 0.05). On the other hand, the impact of region was unclear: (1) living in the suburbs led to a better quality of life in the PF, GH, and VT dimensions, while the impact was negative in the SF, RE, and MH dimensions; and (2) living in rural regions resulted in a better quality of life in the RP, BP, and VT dimensions, but the impact was negative in the MH dimension with a high standardized regression coefficient.

**Table 4:** Standardized regression coefficients of the influence of risk factors on quality of life resulted from multivariate stepwise regression.

Factor	PF	RP	BP	GH	VT	SF	RE	MH
Region								
Urban								
Suburban	0.16*	-0.06		0.10*	0.18*	-0.08*	-0.10*	-0.08*
Rural		0.10*	0.14*		0.10*			-0.25*
Gender			0.05					
Current job				-0.08*	-0.10*	-0.07*		
Age	-0.27*		-0.08*	-0.26*	-0.15*			
Current marital status					0.08*			0.08*
Highest level of education		0.11*						
Total income of family/month						0.05	0.07*	0.15*
Frequency of activities	0.10*	0.05		0.10*	0.13*			0.10*
BMI				0.05				
Chronic diseases	-0.16*	-0.21*	-0.15*	-0.23*	-0.17*	-0.16*	-0.21*	-0.08*

*P-value < 0.05

Variable coding: Region (suburban = 1, other = 0; rual = 1, other = 0), gender (male = 1, female = 0), current job (manual worker = 1, office worker = 0), current martial status (unmarried = 1, married = 0), highest level of education (primary education and lower = 1, secondary/high education = 2, university education and higher = 3), family monthly income (less than 2000 Yuan = 1, 2000–4999 Yuan = 2, more than 5000 Yuan = 3), frequency of activities (never = 1, less than 4 times/month = 2, at least 1 time/week = 3, at least 1 time/day = 4), chronic disease (yes = 1, no = 0).

## Discussion

Quality of life is a study area that has attracted increasing interests over the past two decades. SF-36 has been used as an instrument for assessing quality of life world-wide. Normative data have also been obtained in many countries [25,26]. In mainland China, the Mandarin SF-36 has been used in a few surveys to assess the quality of life of general population and the population with special chronic diseases [7-10]. Our survey evaluated the feasibility of using the Mandarin SF-36 for investigating health related quality of life in the population of Shanghai, China.

Statistical analyses used in this study included split-half reliability coefficient, ICC, paired-sample t test for the difference between the test and retest scores, and Cronbach's α. The results indicated that SF-36 was quite stable for the purposes of the study with a good internal consistency.

In particular, the SF dimension had the lowest Cronbach's α coefficient in this study, which was consistent with other surveys using the Mandarin version of SF-36 [10,21,27,28]. The SF dimension also had the lowest ICC and split-half reliability, indicating there might be some problems in the conceptualization of social function. Traditionally, Chinese people don't think much about social function, and commonly say little or nothing about how the physical health or emotional problems would interfere with their social activities. In addition, the SF dimension included two questions as follows: (1) "during the past 4 weeks, to what extent has your physical health or emotional problems interfered with your normal social activities with family, friends, neighbors, or groups?", and (2) "during the past 4 weeks, how much of the time has your physical health or emotional problems interfered with your social activities (like visiting with friends, relatives, etc.)?" It appeared that the answers of the two questions had reverse orders, which may lead to the low reliability. The other reason may be the cultural diversity. In China, "social activities", translated as "she hui huo dong", refer to not only the everyday life within a family or one's circle of friends, but also the formal activities with other people such as going to a ballroom dancing event or attending a conference. The misunderstanding may result in the low reliability.

MH and VT also had relatively low reliabilities [[10,21,27], and [28]]. The Cronbach's α coefficients for the VT and MH dimensions were 0.66 and 0.75 in the survey of Hangzhou [10], 0.72 and 0.71 in Sichuan [27], 0.74 and 0.77 in Hong Kong [28], 0.73 and 0.74 in American Chinese [21], and 0.78 and 0.69 in our study, respectively. This may be due to the characteristics of Chinese people since they are not used to talking about their feelings and emotions in public.

Our results indicated a credible construction validity of SF-36 that was consistent with the outcomes of other surveys [10,29]. Factor analysis proved that our results were basically in accordance with the theoretical construction of SF-36. Correlation analysis indicated that each of the 36 items was highly correlated within the hypothesized dimensions, while relatively low correlations were observed between the items and other dimensions.

Therefore, we concluded that SF-36 was acceptable and applicable for evaluating the quality of life in the general population of Shanghai, China. Compared our survey with other studies, American Chinese had the worst quality of life among different Chinese populations. Shanghai population had the best quality of life, even better than American and Canadian [9,10,21-25]. It should be noticed that the other studies in the comparison were undertook much earlier in time than our survey, and China has made impressive progresses in living standard during recent years. Especially, Shanghai is the financial and commercial center of China with the best medical and sanitation conditions. For example, the average life expectancy of the Shanghai population was 81.08 years old in 2007, which is slightly lower than the average life expectancy of Andorra, Macau, Japan, Singapore, San Marino, Hong Kong, and Canada. The infant mortality rate was 3.0‰ and maternal mortality rate was 6.68 deaths per 100,000 live births [30,31]. All these factors may lead to high HRQL in the Shanghai population.

In addition, we found a very interesting fact about the normative values of the SF-36 dimension scores. Although the female had worse HRQL than the male in most subgroups, in some subgroups female did report a better mental health. The same results were found in other Chinese populations [9,10,21,22], a fact which is not usual in non-Chinese population. The outcome indicated the serious mental problems in Chinese men, which might be due to the huge stress in both work and life.

We found that region, gender, current job, current marital status, the highest level of education, total income of family per month, frequency of activities, BMI, and chronic diseases had influences on at least one SF-36 dimension. But drinking and smoking did not significantly affect HRQL. When the interaction effects among these factors were excluded in multivariate regression, some risk factors such as resident region, chronic diseases, current job, frequency of activities, and age had strong influences on three or more SF-36 dimensions, while current marital status, the highest level of education, and total income of family per month affected only one or two of the SF-36 dimensions. These results were analogous to the previous study in Sichuan in which chronic condition, personal income, inhabitant places, age, and educational level were found to be the significant risk factors influencing quality of life, while marital status had impacts on few SF-36 dimensions [32]. All of the SF-36 dimensions were remarkably impaired by chronic diseases. People with chronic diseases had a worse quality of life than those without. It had been considered as the main risk factor impairing quality of life [7,8,32]. The PF, MH, GH, and VT dimensions were highly correlated with frequency of activities. Everyday activities, such as housework and walking, may help to stay healthy. The impact of age on quality of life was also notable. It was evident that the health problems became more and more serious with increasing age.

There are some limitations in this study. Detailed information on non-responders were not collected, we were not sure whether there were differences between responders and non-responders. Although the interviewers received uniform training, there still might be influence of the interviewers' explanation on the results, and it was difficult to evaluate, which was also the limitation of this survey. Migrant workers, who make up a significant portion of the Shanghai population, were unable to be sampled because they remain officially registered in their place of origin. In addition, the sampling in the suburbs should be considered more carefully. Since the 1990s, urban population increased rapidly due to economic development and suburbanization in Shanghai. More and more people settled down in suburban regions, especially the group of white collars [33]. It made the distribution of suburban population more complicated.

## Conclusion

In summary, the Mandarin SF-36 is a valid and reliable questionnaire for evaluating both physical and mental health status. The quality of life in the Shanghai population is quite good compared with those in other Chinese populations. The primary influencing factors are region, chronic diseases, age, and frequency of activities. The key to improving quality of life includes the prevention and control of chronic diseases, and participating in moderate and regular activities. In addition, the elderly people should pay more attention on quality of life.

## Competing interests

The authors declare that they have no competing interests.

## Authors' contributions

RW assisted with the survey, completed the statistical analyses and drafted the manuscript. CW, YFZ, XYY and MJW assisted with the study, and XQM assisted with the survey and data analyses. WBL, ZG and JZ participated in the design of the study. JH conceived of the study, and participated in its design and coordination. All authors read and approved the final manuscript.

## Pre-publication history

The pre-publication history for this paper can be accessed here:



## Supplementary Material

Additional file 1 A health related quality of life survey in the population of Shanghai, China. English and Mandarin Chinese translations of a survey instrument for the HRQL study in China, including general information and SF-36 questionnaire. Click here for file
