# Supplementary material for: Health related quality of life measured by SF-36: a population-based study in Shanghai, China
Source: BMC Public Health. 2008 Aug 19;8:292. doi: 10.1186/1471-2458-8-292 (PMC2527564; doi:10.1186/1471-2458-8-292)
Supplement: Additional file 1 — A health related quality of life survey in the population of Shanghai, China. English and Mandarin Chinese translations of a survey instrument for the HRQL study in China, including general information and SF-36 questionnaire. [file 1471-2458-8-292-S1.doc]

上海预调查

A pilot survey in general population, Shanghai

| Q0001 | 调查点代码:  Surveyed site number: | 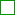[[1]](#footnote-2) |
| --- | --- | --- |
| Q0002 | 调查对象编号:  Respondent ID: | 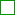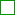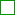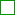 |
| Q0003 | 是初次调查还是再次调查？ 1.初次 2.再次  Is this the initial or retest interview? 1.Initial 2. Retest | 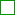 |
| Q0003a | 如果是再次调查，请指明两次调查间的间隔天数：(天)  If retest interview, indicate number of days between initial and retest: (Days) | 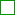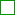 |
| Q0004 | 调查员编号  Interviewer ID | 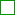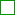 |
| Q0005 | 调查员姓名  Interviewer name | ______________[[2]](#footnote-3) |
| Q0006 | 调查完成日期(年/月/日)  Date of finish(Year/Month/Day) | 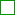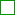/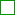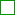/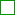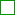 |
| Q0007 | 调查结果： 1.完成 2.部分完成 3.失访  Final result 1. Finished 2. Part finished 3. Missing | 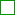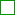 |
| Q0008 | 督导员审核日期(年/月/日)  Date of audit(Year/Month/Day) | 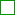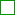/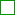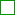/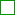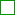 |
|  | 督导员签字  Signature of Supervisor | ______________ |

| 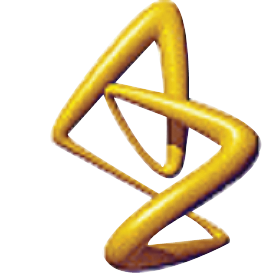 | AstraZeneca R&D Mölndal |
| --- | --- |
| 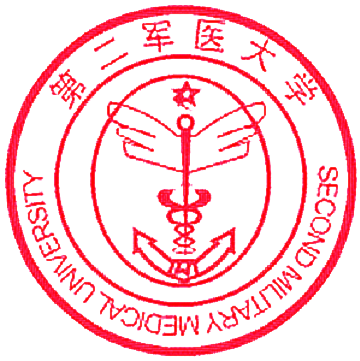 | **第二军医大学卫生统计学教研室** |

2005.10

知情同意书

Consent Form

**尊敬的参与者：**

**您好。**

**您已被随机地选为本次研究的调查对象，我们将对您作有关调查。本次研究是阿斯利康国际药业公司与第二军医大学卫生统计学教研室联合开展的一项预调查研究，由专业人员进行调查。您所提供的信息仅用于了解影响您健康的一些问题，整个调查约需30分钟。**

**您提供的信息不会泄漏给任何人，只用于研究目的。您的姓名、地址和其他个人信息将从调查表中删去，仅用代码与您的姓名和您的回答相联系，单从调查表不能认出是您本人。调查组在必要时可能会再次与您联系。**

**您的参与是自愿的，并且可以在调查过程中退出。您可以拒绝回答调查问卷中的任何一个问题。**

**在调查过程中，如果您有任何问题，可直接问调查员或直接与第二军医大学卫生统计学教研室的督导员联系。**

**签字表示您已了解您在本次调查中需要做的事情并愿意参加本次调查。**

**Dear Participant,**

**You have been randomly selected to be part of this survey and we would, therefore, like to interview you. This survey is a pilot study conducted by the AstraZeneca International Pharmaceutical Company and Department of Health Statistics, Second Military Medical University (SMMU), and will be carried out by professional interviewers. The information you provide will only be used to understand the main things that affect your health. The interview will take approximately 30 minutes.**

**The information you provide is totally confidential and will not be disclosed to anyone. It will only be used for research purposes. Your name, address, and other personal information will be removed from the questionnaire, and only a code will be used to connect your name and your answers without identifying you. The Survey Team may contact you again only if it is necessary to complete the information on the survey.**

**Your participation is voluntary and you can withdraw from the survey after having agreed to participate. You are free to refuse to answer any question that is asked in the questionnaire.**

**If you have any questions about this survey you may ask interviewer or contact Principal Investigator of Department of Health Statistics, SMMU..**

**Signing this consent indicates that you understand what will be expected of you and are willing to participate in this survey.**

**调查对象签名： 日期：**

**Sign of respondent:** ______________  **Date:**
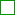

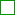
/
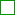

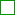
/
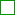

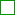


**联系电话：___________________**

第一部分：基本情况

Part I: General Information)

|  |  | | 填写答案处  Answer |
| --- | --- | --- | --- |
| Q1001 | 姓名(请用汉语拼音填写) Full name (Please fill with English) | | __________[[3]](#footnote-4) |
| Q1002 | 性别: 1-女性 2-男性 Sex:: 1-Female 2-Male | | 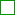[[4]](#footnote-5) |
| Q1003 | 年龄 (岁) Age (yrs) | | 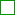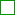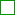 |
| Q1003a | 如果记不清具体年龄，请选择您所在的年龄段：  1. 18~19岁 2. 20~29岁 3. 30~39岁  4. 40~49岁 5. 50~59岁 6. 60~69岁 7. 70+  If you don't know/don’t want to tell me your age could you tell me the age range:  1. 18~19yrs 2. 20~29yrs 3. 30~39yrs  4. 40~49yrs 5. 50~59ysr 6. 60~69ysr 7. 70+ | | 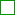 |
| Q1004 | 体重(公斤) Weight(kg) | | 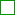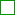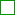 |
| Q1005 | 身高(厘米) Height(cm) | | 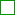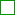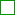 |
| Q1006 | 婚姻状况: 1-已婚 2-未婚  Current marital status: 1- Married 2-Unmarried | | 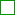 |
| Q1007 | 最高学历: 1-小学以下 2-小学 3-初中 4-高中 5-大专/大学 6-硕士 7-博士  The highest level of education: 1-Less than primary school 2-Primary school completed 3-Secondary school completed 4-High school (or equivalent) completed 5-College / pre-university / University completed 6-Master degree 7-Doctor degree | | 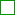 |
| Q1008 | 您总共接受过多少年学校教育(包括复读时间)？  How many years of school education have you completed (including repeated grades)? | | 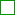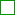 |
| Q1009 | 民族(请用汉语拼音填写) Nationality (Please fill with English) | | ___________ |
| Q1010 | 职业: 1-公务员 2-专业技术人员(军人除外) 3-工人  4-农、林、牧、渔业从业者 5-服务行业人员 6-在校学生 7-军人  Current job: 1-Government employee 2-Professional or technician (excluding armed forces) 3-Blue-collar worker 4-Agricultural or fisheries Worker  5-Personal services, marketing, or sales 6-Student in school 7-Armed forces | | 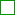 |
| Q1011 | 家庭月收入: 1-1999元以下 2-2000~4999元 3-5000~9999元 4-10000元以上  Total income of family per month: 1-less than 1999 Yuan 2-2000~4999 Yuan  3-5000~9999 Yuan 4-10000 Yuan or above | | 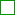 |
| Q1012 | 吸烟情况:  1-不吸 2-1~5支/天 3-6~10支/天  4-11~15支/天 5-16~20支/天 6-21支/天 以上  Do you currently smoke?  1-No 2-1~5pieces/day 3-6~10pieces/day  4-11~15pieces/day 5-16~20pieces/day 6-21pieces/day or above | | 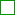 |
| Q1012a | 如果您吸烟，请说明您大概吸了多少年了？  If you smoke, for how many years are you have been smoking? | | 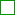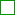 |
| Q1013 | 饮酒情况: 1-不饮酒 2.-每月少于4次 3-每周至少一次 4-每天至少一次  Do you currently drink?: 1-No 2-Less than 4 times per month  3-At least 1 time per week 4-At least 1 time per day | | 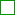 |
| Q1013a | 如果您饮酒，请说明您大概饮酒多少年了？  If you drink, for how many years are you have been drinking? | | 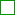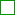 |
| Q1014 | 请选择您平时进行体育运动的频率:  1-不运动 2-每月少于4次 3-每周至少一次 4-每天至少一次  How often do you take physical activities?  1-Never 2- Less than 4 times per month  3-At least 1 time per week 4-At least 1 time per day | | 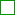 |
| Q1015 | 总体来说，您的健康状况: 1-非常好 2-好 3-一般 4-差 5-很差  In general, how would you rate your health? 1-Very good 2-Good 3-Moderate 4-Bad 5-Very Bad | | 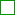 |
| Q1016 | 总体来说，您从事日常活动的能力: 1-非常好 2-好 3-一般 4-差 5-很差  Overall, how would you rate your daily activity? 1-Very good 2-Good 3-Moderate 4-Bad 5-Very Bad | | 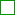 |
| Q1017 | 总体来说，您从事工作的能力: 1-非常好 2-好 3-一般 4-差 5-很差  Overall, how would you rate your daily work?: 1-Very good 2-Good 3-Moderate 4-Bad 5-Very Bad | | 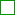 |
| Q1018 | 总体来说，您的精神状态: 1-非常好 2-好 3-一般 4-差 5.-很差  Overall, how would you rate your spirit? 1-Very good 2-Good 3-Moderate 4-Bad 5-Very Bad | | 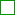 |
| Q1019 | 总体来说，您的社会交往情况: 1-非常好 2-好 3-一般 4-差 5-很差  Overall, how would you rate your interpersonal activities? 1-Very good 2-Good 3-Moderate 4-Bad 5-Very Bad | | 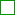 |
| **Q1020** | | **你是否被医生诊断为以下情况/疾病？（如果是，最早做出这一诊断是在您多大年纪时？）**  **Have you been diagnosed by a physician with any of the following conditions/diseases?？（If yes, at which age was it first diagnosed）** | **年龄（岁）**  **Age（yrs）** |
| Q1020a | | 高血压 Hypertension | 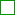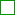 |
| Q1020b | | 缺血性心脏病 Ischemic heart disease | 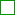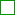 |
| Q1020c | | 脑血管功能异常 Cerebrovascular disorder | 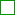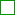 |
| Q1020d | | 糖尿病 Diabetes | 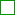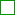 |
| Q1020e | | 慢性阻塞性肺病 COPD | 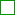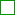 |
| Q1020f | | 哮喘 Asthma | 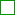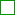 |
| Q1020g | | 肾功能异常 Renal disorder | 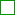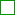 |
| Q1020h | | 肝功能异常 Liver disorder | 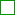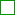 |
| Q1020i | | 风湿性关节炎 Rheumatoid arthritis | 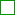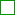 |
| Q1020j | | 骨关节炎 Osteoarthritis | 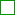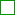 |
| Q1020k | | 焦虑症 Anxiety | 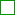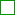 |
| Q1020l | | 抑郁症 Depression | 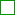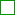 |
| **Q1021** | | **您现在正在服下列药吗？如果有，请在后面写出已服用了多久**  **Are you taking following drugs currently? If yes, please write the duration that you have taken them?** | **时间(月)**  **Duration(months)** |
| Q1021a | | 皮质类固醇 Corticosteroids | 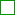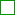 |
| Q1021b | | 荷尔蒙替代品 Hormone replacement | 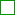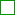 |
| Q1022 | | 现在服用的其它药物一，名称： Others (name)： | __________[[5]](#footnote-6) |
| Q1022a | | 服用了多少时间(月) Duration of treatment(months) | 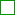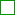 |
| Q1023 | | 现在服用的其它药物二，名称： Others (name)： | ___________ |
| Q1023a | | 服用了多少时间(月) Duration of treatment(months) | 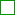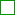 |
| Q1024 | | 现在服用的其它药物三，名称： Others (name)： | ___________ |
| Q1024a | | 服用了多少时间(月) Duration of treatment(months) | 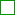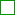 |

第二部分：SF-36健康调查问卷

Part Ⅱ: SF-36

本调查涉及你对自身健康的观点。这些信息将有助于追踪你从事日常活动的能力及自身感觉。请回答所有问题，在方框内填下你所选择的的数字。如果你对答案不确定，请给出你认为最接近的答案。

This survey asks for your views about your health. This information will help keep track of how you feel and how well you are able to do your usual activities.Answer every question by marking the answer as indicated. If you are unsure about how to answer a question, please give the best answer you can.

| Q6001 | 总的来说，你认为你的健康状况： 1-棒极了 2-很好 3-好 4-过得去 5-糟糕  In general, would you say your health is: 1-Excellent 2-Very good 3-Good 4-Fair 5-Poor | 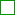 |
| --- | --- | --- |
| Q6002 | 与一年前相比，你如何评价现在的健康状况？  1-比一年前好多了 2-比一年前好一点 3-和一年前差不多  4-比一年前差一点 5-比一年前差多了  Compared to one year ago, how would you rate your health in general now?  1-Much better now than one year ago 2-Somehwat better now than one year ago 3-About the same as one year ago 4-Somewhat worse  now than one year ago 5-Much worse now than one year ago | 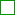 |
| **Q6003** | 下列项目是你平常在一天中可能做的事情。你现在的健康**限制**你从事这些活动吗？如果是的话，程度如何？  **选择项:**  **1-是，很受限 2-是，稍受限 3-不，完全不受限**  The following items are about activities you might do during a typical day. Does your health now limit you in these activities? Is so, how much?  **Selection:**  **1-Yes, limited a lot 2-Yes, limited a little 3-No, not limited at all** |  |
| Q6003a | **高强度活动，**如跑步、举重物、参与剧烈运动：  Vigorous activities, such as running, lifting heavy objects, participating in strenuous sports: | 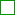 |
| Q6003b | **中等度活动，**如移动桌子，推动真空吸尘器（或拖地板）、打保龄球、打高尔夫球（或打太极拳）：  Moderate activities, such as moving a table, pushing a vacuum cleaner, bowling, or playing golf: | 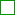 |
| Q6003c | 举或搬运杂物：  Lifting or carrying groceries: | 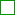 |
| Q6003d | 爬**数**层楼梯：  Climbing several flights of stairs: |  |
| Q6003e | 爬**一**层楼梯：  Climbing one flight of stairs: |  |
| Q6003f | 弯腰、屈膝：  Bending, kneeling, or stooping: |  |
| Q6003g | 步行**1500米以上**：  Walking more than a mile: |  |
| Q6003h | 步行**几个路口**：  Walking several blocks: |  |
| Q6003i | 步行**一个路口**：  Walking one block: |  |
| Q6003j | 自己洗澡或穿衣：  Bathing or dressing yourself: |  |
| **Q6004** | **在过去4周，你是否因为生理健康原因，在工作或从事其他日常活动时有下列问题？**  **During the past 4 weeks, have you had any of the following problems with your work or other regular daily activities as a result of your physical health?** |  |
| Q6004a | 减少了工作或从事其他活动的**时间**：1-是 2-否  Cut down on the amount of time you spent on work or other activities: 1-Yes 2-No |  |
| Q6004b | 减少了工作**量**或活动量：1-是 2-否  Accomplished less than you would like：1-Yes 2-No |  |
| Q6004c | 从事工作或其他活动的**种类**受限：1-是 2-否  Were limited in the kind of work or other activities.1-Yes 2-No |  |
| Q6004d | 从事工作或其他活动有**困难**（例如，费劲）：1-是 2-否  Had difficulty performing the work or other activities (for example, it took extra effort): 1-Yes 2-No |  |
| **Q6005** | **在过去4周，你是否因为任何情感问题（如感到抑郁或焦虑），在工作或从事其他日常活动时有下列问题？**  **During the past 4 weeks , have you had any of the following problems with your work or other regular daily activities as a result of any emotional problems (such as feeling depressed or anxious)** |  |
| Q6005a | 减少了工作或从事其他活动的**时间**：1-是 2-否  Cut down on the amount of time you spent on work or other activities: 1-Yes 2-No |  |
| Q6005b | 减少了工作**量**或活动**量**：1-是 2-否  Accomplished less than you would like：1-Yes 2-No |  |
| Q6005c | 不能象平常那么专心地从事工作或其他活动：1-是 2-否  Didn’t do work or other activities as carefully as usual：1-Yes 2-No |  |
| Q6006 | 在过去4周，你的生理健康或情感问题在何种程度上干扰了你与家人、朋友、邻居、或团体的正常社会活动？ 1-完全没有 2-轻度 3-中度 4-重度 5-极度  During the past 4 weeks, to what extent has your physical health or emotional problems interfered with your normal social activities with family, friends, neighbors, or groups?  1-Not at all 2-Slightly 3-Moderately 4-Quite a bit 5-Extremely |  |
| Q6007 | 在过去4周，你经受了多少躯体疼痛？  1-完全没有 2-很轻微 3-轻微 4-中等 5-严重 6-极严重  How much bodily pain have you had during the past 4 weeks?  1-None 2-Very mild 3-Mild 4-Moderate 5-Severe 6-Very severe |  |
| Q6008 | 在过去4周，疼痛在多大程度上干扰了你的正常工作（包括户外工作和家务劳动）？  1-完全没有 2-一点点 3-中度 4-重度 5-极度  During the past 4 weeks, how much did pain interfere with your normal work (including both work outside the home and housework)?  1-Not at all 2-A little bit 3-Moderately 4-Quite a bit 5-Extremely |  |
| **Q6009** | 这些问题将问及你在过去4周的感觉和情感体验。对每一问题，请给出与你想法最接近的一个答案。在过去4周，**有多少时间**…  **选择项:**  **1-所有时间 2-绝大多数时间 3-很多时间 4-一些时间 5-一点时间 6-没有时间**  These questions are about how you feel and how things have been with you during the past 4 weeks. For each question, please give the one answer that comes closest to the way you have been feeling. **How much of the time during the past 4 weeks**  **Selection:**  **1- All of the time 2-Most of the time 3-A good bit of the time**  **4-Some of the time 5-A little of the time 6-None of the time** |  |
| Q6009a | 你觉得干劲十足？  Did you feel full of pep? |  |
| Q6009b | 你是一个非常紧张的人？  Have you been a very nervous person? |  |
| Q6009c | 你感到情绪低落、沮丧，怎么也快乐不起来？  Have you felt so down in the dumps that nothing could cheer you up? |  |
| Q6009d | 你觉得平静、安适？  Have you felt calm and peaceful? |  |
| Q6009e | 你觉得精力旺盛？  Did you have a lot of energy? |  |
| Q6009f | 你感到闷闷不乐、心情忧郁？  Have you felt downhearted and blue? |  |
| Q6009g | 你觉得累极了？  Did you feel worn out? |  |
| Q6009h | 你是一个快乐的人？  Have you been a happy person? |  |
| Q6009i | 你觉得疲劳？  Did you feel tired? |  |
| Q6010 | 在过去4周，有多少时间你的社会活动（如访问朋友，亲戚等）受你的生理健康或情感问题的影响动？1-所有时间 2-绝大多数时间 3-一些时间 4-一点时间 5-没有时间  During the past 4 weeks, how much of the time has your physical health or emotional problems interfered with your social activities (like visiting with friends, relatives, etc.)?  1- All the time 2-Most of the time 3- Some times 4-A little time 5-None of the time |  |
| **Q6011** | **下列每一种情形与你实际情况符合的程度如何?**  **How TRUE or FALSE is each of the following statements for you?** |  |
| Q6011a | 和其他人相比，我似乎更容易生病：  1-全部符合 2-大部分符合 3-不知道 4-大部分不符合 5-全部不符合  I seem to get sick a little easier than other people  1-Definitely true 2-Mostly true 3-Don’t know 4-Mostly false 5-Definitely false |  |
| Q6011b | 我和我认识的人一样健康：  1-全部符合 2-大部分符合 3-不知道 4-大部分不符合 5-全部不符合  I am as healthy as anybody I know  1-Definitely true 2-Mostly true 3-Don’t know 4-Mostly false 5-Definitely false |  |
| Q6011c | 我预计我的健康状况将变得更差：  1-全部符合 2-大部分符合 3-不知道 4-大部分不符合 5-全部不符合  I expect my health to get worse  1-Definitely true 2-Mostly true 3-Don’t know 4-Mostly false 5-Definitely false |  |
| Q6011d | 我的身体棒极了：  1-全部符合 2-大部分符合 3-不知道 4-大部分不符合 5-全部不符合  My health is excellent  1-Definitely true 2-Mostly true 3-Don’t know 4-Mostly false 5-Definitely false |  |

1. 每个空格处填写一个数字 [↑](#footnote-ref-2)
2. 请用大写英文字母填写 [↑](#footnote-ref-3)
3. 横线处请用大写字母填写。 [↑](#footnote-ref-4)
4. 每个空格处填写一个数字，带有小数者请四舍五入。 [↑](#footnote-ref-5)
5. 如不知英文名，可用中文填写。 [↑](#footnote-ref-6)
